# Supplementary material for: Resentful, Resigned and Respectful: Opioid Analgesics, Pain and Control, a Qualitative Study
Source: Pharmacy (Basel). 2025 Feb 11;13(1):25. doi: 10.3390/pharmacy13010025 (PMC11858891; doi:10.3390/pharmacy13010025)
Supplement: Supplementary file 1 [file pharmacy-13-00025-s001.zip › pharmacy-3432036-supplementary.pdf]

Examples of Qualitative Interview Questions

1. Please could you summarise your use of current opiate medication in terms of the medicines you take and for what conditions and symptoms.
    a. Prompt in relation to medical condition/symptoms
    b. Whether primary or secondary care
2. Who has prescribed these for you?
    a. Ask for further details about continuity of care, type of prescriber etc.
3. Please briefly indicate what previous medicines have you taken (for pain relief).
4. To what extend do you feel your current opiate medication is appropriate for your condition and symptoms. Prompt if necessary about:
    a. whether the opioid (or other treatments) have managed to control the pain
    b. if there have been any side effects
5. Do you have any concerns about the opiate medication you are currently taking?
    a. If so, what are these and please describe them.
    b. Follow-up to 5) and 5a): if yes: have you raised any of these concerns with anyone and if so, what happened?
6. Do you feel you had sufficient information about your current opiate medication?
    a. Please describe why in more detail.
7. Are you aware of the potential for some individuals to become dependent or addicted to opiate medication?
    a. If yes, please can you indicate what you understand by this and how you became aware of this.
8. Do you feel you ever have been or currently are addicted to an opiate pain medication?
    a. If so, please could you describe why you think this and your experience.
    b. Follow-up to 8a): if yes, what help or treatment was sought and was this beneficial?
9. Have you ever felt any anger or mistrust towards a current or previous prescriber/doctor?
    a. If so, please give details of why you felt this and how this arose and if it was resolved.
10. Have you obtained pain medication from other sources apart from prescription?
    a. If so, please say where and why this source was used [prompt, from pharmacy purchase, internet, another person's medicines].
